# Mechanical and Dynamic Performance of a High-RAP Half-Warm Asphalt Polymeric Composite for Rapid Pavement Repair

**DOI:** 10.3390/polym18060676

**Published:** 2026-03-11

**Authors:** Shanelle Aira Rodrigazo, Ik Hyun Hwang, Junhwi Cho, Ilhwan You, Kwan Kyu Kim, Jaeheum Yeon

**Affiliations:** 1 Interdisciplinary Program in Earth Environmental System Science & Engineering, Kangwon National University, Chuncheon 24341, Republic of Korea; rodrigazo.shanelleaira@kangwon.ac.kr (S.A.R.); xnxlwnsgnl@kangwon.ac.kr (J.C.); 2 City of Tech Co., Ltd., Yongin 16801, Republic of Korea; cityoftech@naver.com; 3 Department of Rural Construction, Jeonbuk National University, Jeonju 54896, Republic of Korea; ih-you@jbnu.ac.kr; 4 Korea Conformity Laboratories, Chuncheon 24341, Republic of Korea; 5 Department of Regional Infrastructure Engineering, Kangwon National University, Chuncheon 24341, Republic of Korea

**Keywords:** pothole, half-warm mix asphalt, reclaimed asphalt pavement, low-temperature additive, pavement maintenance

## Abstract

High reclaimed asphalt pavement (RAP) half-warm mix asphalt (HWMA) mixtures provide a low-energy alternative for pavement repair but often suffer from insufficient binder activation and reduced mechanical performance at low production temperatures. This study develops a high-RAP (73.8%) half-warm repair mixture using a dual-additive system comprising a rejuvenator and a low-temperature composite additive. The mixture was designed to enable effective mixing and compaction at temperatures as low as 60 °C. The optimized formulation achieved a 5.84 kN Marshall stability, 7.0% voids in total mixture, 80% retained Marshall stability after moisture conditioning, and approximately 1100 passes/mm dynamic stability. Temperature sensitivity analysis showed that stability increased from 4.50 kN at 50 °C to 9.20 kN at 90 °C with corresponding VTM reduction from 15.2% to 4.8%. The results demonstrate that a high-RAP HWMA repair mixture can satisfy mechanical and durability requirements while being produced at substantially reduced temperatures, supporting practical and sustainable pavement maintenance applications. The study further provides mixture-scale evidence that a dual-additive strategy can stabilize high-RAP mixtures under very low half-warm production temperatures (≈60–70 °C), which are representative of rapid repair conditions and remain insufficiently investigated in existing WMA–RAP research.

## 1. Introduction

Pothole damage remains one of the most persistent and costly challenges in road maintenance, compromising pavement serviceability and traffic safety. Timely and durable repairs are essential, particularly in urban areas and cold climates where freeze–thaw cycles accelerate deterioration [1]. While hot-mix asphalt (HMA) is the benchmark for permanent repairs due to its superior mechanical performance, its production requires high temperatures (150–170 °C) and access to centralized plants [2], limiting its practicality for small-scale, scattered, or cold-weather applications [3,4]. Cold-mix asphalt (CMA), in contrast, can be stored and placed without heating [3], making it widely used for emergency patching. However, CMA’s slow strength gain, high air voids, and poor moisture resistance result in short service lives and frequent rework [5,6].

Warm mix asphalt (WMA) has emerged as a promising middle ground. By incorporating chemical additives or foaming, WMA allows mixing and compaction at 20–40 °C below HMA [7,8], with half-warm variants (HWMA) operating at even lower temperatures (~70–100 °C) [9]. These systems offer substantial energy and emissions reductions while extending the paving window into colder seasons—key for rapid patching. However, lowering the production temperature may compromise binder activation, coating, and aggregate drying [10,11], often resulting in excessive air voids, poor cohesion, and increased moisture sensitivity [12].

Simultaneously, increasing reclaimed asphalt pavement (RAP) content aligns with sustainability goals by reducing demand for virgin materials [13,14]. However, high-RAP mixtures present challenges due to the stiff, aged binder, which alters the viscoelastic balance of the composite matrix and typically requires rejuvenators to restore ductility and flow characteristics [15]. Rejuvenator performance is strongly dependent on temperature and activation time. Under HWMA conditions (≤100 °C) and short mixing times typical of patching, insufficient binder diffusion can hinder proper blending and degrade mechanical performance [16,17].

Binder ageing in RAP is primarily governed by thermo-oxidative reactions, volatilization of light fractions, ultraviolet exposure, and long-term environmental conditioning, all of which progressively alter the binder’s chemical composition and rheological behavior. These processes increase stiffness, reduce ductility, and shift the internal balance of binder fractions, leading to limited molecular mobility and reduced blending efficiency with virgin binder during mixing. Under half-warm and short-duration mixing conditions, such ageing effects become more critical, as insufficient thermal energy further restricts binder diffusion and activation, thereby exacerbating workability and cohesion deficiencies in high-RAP mixtures. Previous studies have also linked insufficient ageing resistance of bitumen to the development of cracks, ruts, and potholes, emphasizing that technological and environmental ageing processes degrade binder quality and weather resistance, thereby accelerating coating deterioration and pavement distress [18].

To address the limitations of high-RAP mixtures at reduced temperatures, recent studies have increasingly explored combined additive strategies that integrate recycling agents with structural modifiers. Aromatic oil-based rejuvenators are widely used to soften oxidized binders and enhance diffusion and blending efficiency, whereas stiffening modifiers such as asphaltites and polymer-based additives can improve rutting resistance and structural stability of recycled mixtures. Resin-based components and adhesion promoters have also been reported to enhance binder–aggregate interaction and moisture resistance, particularly under low-temperature compaction conditions. Accordingly, dual-additive systems are expected to provide complementary effects by simultaneously improving binder mobility, coating behavior, and mixture stability, although such functional roles are interpreted based on prior literature rather than directly measured mechanisms in the present study. In high-RAP mixtures, supplementary additives such as plasticizers, process oils, recycled polymers (e.g., PET), and mineral modifiers are typically incorporated to compensate for the stiffness and limited mobility of aged binder. Plasticizing and resin-based components primarily enhance ductility and workability and may improve adhesive properties at the binder–aggregate interface [19], whereas rigid modifiers (e.g., gilsonite or polymeric inclusions) are expected to contribute to stiffness recovery and deformation resistance. Recent studies have also explored plastic-based pavement materials without conventional asphalt binder, such as stamp sand–acrylonitrile styrene acrylate waste composites, which demonstrated promising rutting and moisture resistance but lower fracture energy compared with asphalt mixtures, indicating their potential as alternative repair materials while highlighting limitations in cracking performance [20].

Although prior studies have explored rejuvenator types, RAP content, and mixing temperatures, most were developed for full-depth paving with moderate RAP levels (≤50%) and production temperatures typically above 100 °C. Zaumanis et al. (2014) [21] showed that high RAP mixtures require careful binder activation to maintain performance, while Yousefi et al. (2021) [22] reported that the mechanical behavior of WMA–RAP mixtures is highly sensitive to recycling agent type, dosage, and binder ageing condition. Subsequent studies highlight the importance of dosage control. Jiang et al. (2023) [23] demonstrated that rejuvenator dosage strongly influences the rheological recovery of aged binders, with increasing content enhancing penetration and ductility and lowering softening point, underscoring the necessity of optimum dosage selection. At the mixture level, Liao et al. (2023) [24] showed that bio-based rejuvenators can increase rutting and stripping susceptibility in high-RAP mixtures under high-temperature performance conditioning, suggesting that binder softening must be carefully balanced when designing recycled mixtures. When considering low-temperature recycling, Pasandín et al. (2020) [25] indicated that fully recycled HWMA systems often exhibit performance trade-offs compared to mixtures containing virgin materials, and Liu et al. (2024) [26] emphasized that RAP preheating temperature plays a critical role in activating aged binder and enhancing its mobilization. Nevertheless, several studies have predominantly focused on binder-scale rejuvenation mechanisms and rheological restoration [27,28]. In contrast, mixture-scale studies indicate that the performance of rejuvenated RAP mixtures is strongly governed by rejuvenator dosage and production temperature. For example, Zhang et al. (2021) [29] showed that excessive biobinder dosage reduced mixture strength, while higher production temperature improved blending and resistance to rutting and moisture damage.

Despite recent progress, there remains limited quantitative evidence regarding the mechanical and dynamic performance of high-RAP HWMA composite systems produced at very low temperatures and short mixing times, which are representative of practical repair operations [30,31,32]. While some studies have investigated HWMA with high RAP content for surface layers [33] or explored thermomechanical healing approaches involving microwave heating for recycled HWMA materials [34], they have not addressed small-scale patching scenarios or the combined constraints typical of maintenance operations, including limited mixing duration, reduced production temperature, and early traffic loading. Moreover, existing WMA and RAP studies predominantly focus on plant-produced mixtures for paving applications rather than rapid repair contexts, and often employ moderate RAP contents and higher processing temperatures.

For clarity of terminology, the term bitumen in this study refers to the asphalt binder, while asphalt mixture (asphalt concrete) denotes the composite material consisting of mineral aggregates, filler, and binder. The term coating refers to the binder or mastic film covering aggregate particles during mixing.

This study investigates a high-RAP half-warm asphalt composite designed for pothole repair applications incorporating high RAP content, formulated with a dual-additive system comprising a rejuvenator and a low-temperature agent. The objective is to evaluate whether this formulation enables effective mixing, coating, and compaction at production temperatures as low as 60 °C, while maintaining durability and moisture resistance under field-representative repair conditions. Laboratory testing examines how reduced processing temperature and short mixing duration influence binder activation, composite structure, and interfacial cohesion, and how these structural attributes influence performance outcomes, including Marshall stability, flow, moisture susceptibility, and rutting resistance. In contrast to conventional WMA and RAP studies that typically employ moderate RAP contents and higher production temperatures, this work systematically evaluates a polymer-modified high-RAP composite under very low half-warm processing conditions (60–70 °C) representative of rapid repair scenarios, thereby providing mixture-scale evidence on the stabilization of highly aged binder systems through a complementary dual-additive modification strategy. The findings further establish a practical and scientific basis for the design of low-temperature, high-RAP repair materials and support future mechanistic and field-scale investigations of composite additive systems in sustainable pavement maintenance.

## 2. Materials and Methods

### 2.1. Materials

#### 2.1.1. RAP

The core material for the HWMA developed in this study was RAP, sourced from previously milled asphalt layers. The RAP was processed to meet a dense-graded profile with a nominal maximum aggregate size of 12.5 mm, aligning with conventional surface course specifications for pavement repair. This RAP contributed both aged binder and mineral aggregates, making it critical to characterize its properties before mix design.

**Figure 1:**
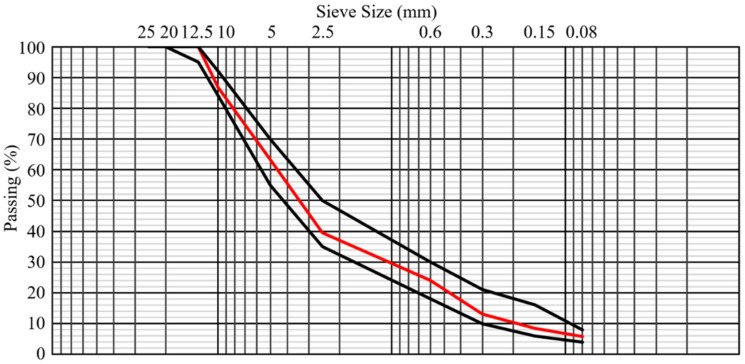
Combined aggregates gradation curve.

To complete the aggregate skeleton and achieve the target gradation, virgin aggregates were used in conjunction with RAP. These aggregates were sourced from locally available crushed stone and separated into coarse (5–12.5 mm) and fine (passing 5 mm) fractions. The gradation of the virgin aggregates was selected to complement the RAP material and to ensure that the combined aggregate blend fell within the specification envelope for dense-graded surface course mixtures. The gradation data for both RAP and virgin aggregates are presented in Table 1, while the resulting combined gradation curve is shown in Figure 1.

To evaluate the state of the aged binder, recovery was performed via solvent extraction in accordance with AASHTO T164 [35], followed by binder separation and viscosity testing. The absolute viscosity of the recovered RAP binder was determined to be approximately 3920.3 Pa·s at 60 °C, indicating severe oxidative ageing and insufficient flowability for reuse without modification. The RAP contained 4.3% asphalt binder by its own weight. When RAP constituted 75% of the aggregate portion of the mix, this resulted in an effective binder contribution of approximately 3.2% by total mixture weight. However, due to severe oxidative ageing of the RAP binder, this amount alone was insufficient to ensure adequate workability, requiring supplementation with virgin asphalt and chemical rejuvenation.

#### 2.1.2. Binder, Rejuvenator, and Additives

**Figure 2:**
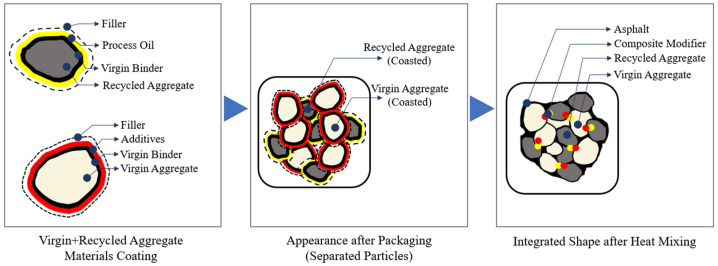
Road maintenance materials composition diagram for pothole.

A composite binder modification strategy was employed to restore the performance characteristics of the aged RAP binder and facilitate HWMA production at reduced temperatures. The virgin binder used in this study was a PG 64-22 paving-grade asphalt, commonly applied in moderate climates due to its balance between rutting resistance and low-temperature flexibility. This binder was pre-blended with a rejuvenating oil and a composite low-temperature additive to form a unified binder system prior to mixing. A schematic illustration of the overall material composition and the interaction between RAP, virgin aggregates, and the modified binder system is presented in Figure 2. Unless otherwise stated, the following materials were sourced from Sejin General Technology Co., Ltd. (Seoul, Republic of Korea).

Two proprietary additive blends were employed: a composite rejuvenator and a low-temperature facilitating additive. The internal formulations of these additives were supplied as fixed blends in accordance with prior technical specifications; their chemical compositions were not redesigned in the present study. The investigation, therefore, focused on dosage optimizations at the mixture level rather than compositional modification. Although proprietary, the additive classes are consistent with commercially available rejuvenation and warm mix technologies commonly applied in asphalt pavement engineering. Only their functional roles and overall dosages are discussed to ensure methodological transparency and reproducibility of the mix design.

The rejuvenator comprised a proprietary blend primarily containing aromatic process oil and polymer-modified constituents, intended to partially restore the maltene–asphaltene balance of the oxidized RAP binder and to reduce its elevated viscosity. It was incorporated at a fixed dosage of 0.38 wt.% of the total mixture mass in the final mix formulation (see Table 2).

The low-temperature additive was a proprietary facilitation blend composed of viscoelastic modifiers, adhesion-promoting agents, and fluxing components intended to enhance aggregate coating, adhesion, and compaction at reduced production temperatures. The additive was incorporated into the binder system as a preformulated modifier, and its internal composition remained fixed throughout the study. Its primary function was to reduce the effective mixture viscosity during mixing and compaction, thereby improving workability under half-warm production conditions while maintaining adequate mechanical stability (see Table 3).

The rejuvenator and the low-temperature additive were used in combination to modify the binder system. The rejuvenator targeted binder softening and ductility restoration, while the low-temperature additive enhanced coating and compaction efficiency without degrading structural performance. The combined system enabled effective mixture workability and compaction at mixing temperatures as low as 60 °C, supporting energy-efficient HWMA production with high RAP content.

### 2.2. Mixture Preparation

The HWMA was prepared using standard laboratory mixing equipment simulating pugmill conditions, with all materials heated to a reduced mixing temperature range of 60–100 °C. The RAP material was pre-heated to the target temperature to activate the aged binder, after which the additive-modified virgin binder and virgin aggregates were added to the mixer. The blending process continued until all aggregate particles were visibly and uniformly coated.

Marshall test specimens were prepared using standard compaction procedures in accordance with ASTM D1559 [36]. After compaction, the specimens were allowed to cure at ambient laboratory temperature (approximately 20–25 °C) for 16 to 18 h. This curing period ensured that any volatiles present in the rejuvenator or low-temperature additive could evaporate, allowing the binder to achieve its final mechanical properties.

The final mix design included 75% RAP and 25% virgin aggregate by aggregate weight, resulting in a total asphalt binder content of 5.3% by mix weight. Of this, approximately 3.2% was contributed by the aged RAP binder, and 2.1% was newly added binder (including both virgin asphalt and the rejuvenator).

### 2.3. Experimental Program

#### 2.3.1. Mix Design and Binder Optimization

The optimum asphalt content (OAC) was determined using a modified Marshall mix design procedure, with initial binder content calculated using the Asphalt Institute MS-21 method:

(1) $$P_{b}\left(\%\right)=0.035a+0.045b+X_{c}+F,$$

where *a*, *b*, and *c* represent the proportions of coarse, intermediate, and fine aggregate fractions, respectively; *X_c_* is the fines activity factor; and *F* is a correction factor. Based on the aggregate gradation of the blended materials, the estimated total binder demand was calculated as $P_{b}=$ 5.3 wt.% (as shown in Table 4). It should be noted that $P_{b}$ represents the required total effective binder content of the mixture rather than the virgin binder content.

**Figure 3:**
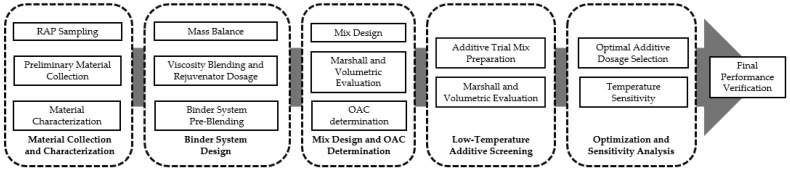
Experimental workflow of high-RAP HWMA mix design and evaluation.

Following the estimation of total binder demand, the mix design and final formulation of the high-RAP half-warm mixture were conducted using a sequential multi-stage procedure (Figure 3). The experimental design was as follows: first, reclaimed materials were gathered and tested to determine their physical properties and binder content. Then, A binder system design was then established through mass balance analysis to account for RAP binder contributions, followed by rejuvenator dosage determination to restore binder workability under half-warm mixing conditions. The Marshall mix design framework was then used to find the OAC after the binder system was stable. Subsequently, trial mixtures were prepared by varying only the low-temperature additive content. The optimal dosage was then determined through sensitivity analysis, and the final mixture composition was reconstructed using full mass normalization to integrate all established design parameters.

The Marshall mix design framework in accordance with ASTM D1559 was adopted to determine OAC based on the estimated binder demand. Trial mixtures were prepared at binder contents of 5.0, 5.3, and 5.6 wt.% and evaluated in terms of bulk density, air voids (VTM), voids in mineral aggregate (VMA), voids filled with asphalt (VFA), Marshall stability, and flow. A target air void content of approximately 4% was adopted in accordance with standard Marshall design practice for asphalt repair mixtures, and the OAC was selected based on volumetric compliance and peak Marshall stability.

For the OAC trial stage, mixture compositions were expressed on a total-mixture mass basis, with the aged asphalt contained in RAP (AP) accounted for separately in the binder mass balance. Accordingly, Table 5 presents the Marshall trial mixtures in which the reclaimed material is reported as the RAP aggregate portion and the inherent aged asphalt content (AP = 3.2 wt.%) is listed independently, consistent with the original Marshall blending procedure used to determine the OAC.

The virgin asphalt content ($P_{nb}$) was determined by a binder mass balance approach using the target total binder content of the mixture ($P_{b}=$ 5.3 wt.%), the asphalt content inherently contained in the RAP obtained from extraction testing ($P_{sb}$ = 4.3 wt.% of RAP), and the virgin aggregate proportion (r = 25%, corresponding to 75 wt.% RAP in the aggregate blend). Based on these parameters, the additional virgin asphalt required to satisfy the target binder demand was calculated as $P_{nb}=$ 2.1 wt.% of the total mixture.

While the binder mass balance established the required binder quantity, the rheological condition of the aged RAP binder necessitated further adjustment to ensure adequate blending and workability under reduced half-warm production temperatures. To evaluate binder stiffness, absolute viscosity testing of the recovered RAP binder was conducted at 60 °C using a vacuum capillary absolute viscosity tester (Cannon Instrument Company, State College, PA, USA) prior to additive incorporation. The recovered binder exhibited elevated viscosity due to oxidative ageing, indicating limited workability and blending potential during low-temperature mixing. Accordingly, a rejuvenator was introduced to condition the binder system and restore practical workability. The rejuvenator content was estimated based on the measured absolute viscosity of the RAP binder and the corresponding viscosity–dosage behavior of the binder system. The required rejuvenator content corresponded to approximately 17.9% of the binder phase, which was subsequently converted to a total mixture basis using the determined virgin binder content (2.1 wt.%), yielding a final rejuvenator dosage of 0.38 wt.% of the mixture. This dosage was then fixed for all subsequent mixture formulations to maintain consistent binder properties during additive optimization and to restore workable binder consistency suitable for half-warm mixing conditions.

After establishing the binder system, the mixture proportions were reconstructed for the additive optimization stage while maintaining constant binder conditions. In this final stage, the reclaimed material was treated as a single integrated component, including its inherent aged binder, and only the low-temperature additive content was varied to produce the trial formulations (Mix A–C) reported in Table 6. These formulations represent the final trial set from which the optimal mix was selected for subsequent performance evaluation.

#### 2.3.2. Specimen Preparation and Curing

Marshall specimens were prepared using a standard Marshall compaction framework in accordance with ASTM D1559, which is commonly applied to asphalt repair and maintenance materials. For all test conditions, specimens were compacted using a Marshall hammer (Heungjin Testing Machine Co., Ltd., Gimpo, Republic of Korea) with 50 blows per face, representing field-relevant compaction effort for pothole repair applications rather than full-scale hot-mix pavement construction.

Following compaction, specimens were allowed to cool and cure at ambient laboratory temperature (approximately 20–25 °C) for 16–18 h prior to testing. This conditioning period allowed stabilization of the binder system and ensured a consistent specimen state before volumetric and mechanical evaluation. The curing duration also permitted the dissipation of any volatile components associated with the rejuvenator or low-temperature additive. Identical compaction and curing conditions were applied to all specimens to maintain consistency across mixtures produced at different binder contents and mixing temperatures.

#### 2.3.3. Performance Test Methods

After finalizing the mix design and establishing the minimum viable production temperature, the performance of the optimized half-warm RAP mixture was evaluated using a series of laboratory tests selected to represent the critical functional requirements of pothole repair materials, including load-bearing capacity, compaction effectiveness, moisture resistance, and resistance to permanent deformation. All tests were conducted on Marshall-compacted specimens following a standard curing period of approximately 16 h at ambient laboratory temperature.

**Figure 4:**
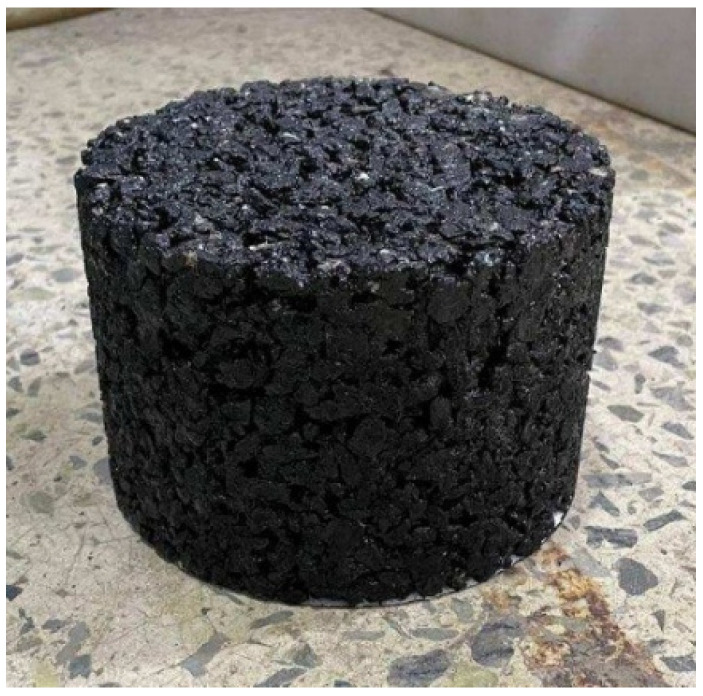
Specimen for the Marshall stability test.

Marshall stability and flow were determined at 60 °C in accordance with ASTM D1559. Specimens were loaded diametrically at a constant deformation rate of 50.8 mm/min until failure, and the peak load was recorded as the Marshall stability, while the corresponding deformation was recorded as the flow value. Marshall stability was used as an indicator of load-bearing capacity, and flow was used to assess mixture ductility and resistance to brittle failure. For asphalt repair applications, a minimum stability criterion of 2.5 kN at 60 °C was adopted. Representative Marshall specimens are shown in Figure 4. Each test condition was evaluated using at least three replicate specimens, and average values are reported.

Volumetric properties were evaluated concurrently with mechanical testing. Air void content was calculated from the measured bulk specific gravity of compacted specimens and the theoretical maximum density, determined in accordance with ASTM D2726 [37] and ASTM D2041 [38], respectively. These measurements were used to assess compaction effectiveness and internal mixture structure, with particular attention to whether reduced production temperatures compromised densification.

Moisture susceptibility was evaluated using retained Marshall stability following water immersion. After initial air curing, a subset of specimens was immersed in water at 60 °C for 48 h, after which Marshall stability was remeasured. Retained stability was calculated as the ratio of wet to dry stability and expressed as a percentage, with a minimum acceptance criterion of 75%. In addition, fractured specimens were visually examined after testing to identify evidence of stripping or loss of binder–aggregate adhesion.

**Figure 5:**
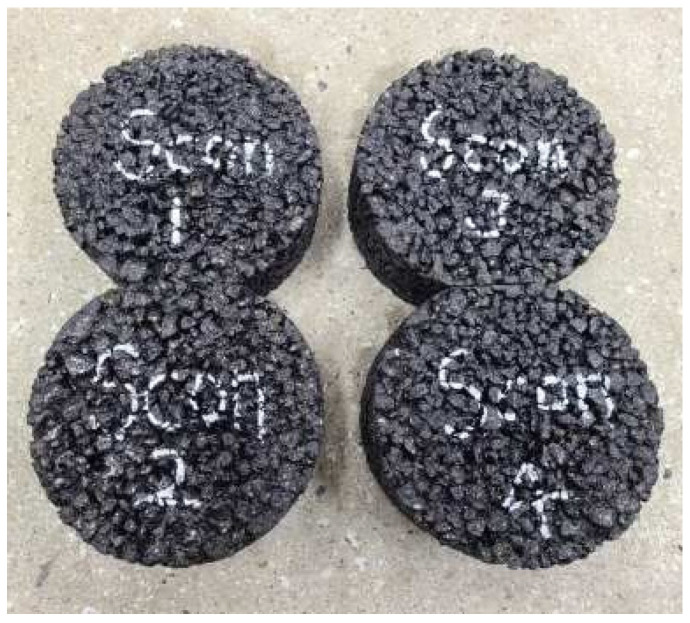
Specimens for the DS test.

Resistance to permanent deformation was assessed using a wheel-tracking test conducted at 60 °C following standard rutting test procedures to simulate severe service conditions associated with early traffic loading. Beam specimens were subjected to repeated wheel passes under controlled loading conditions, and rut depth development was monitored over time. Rutting performance was expressed in terms of dynamic stability, defined as the number of wheel passes required to produce 1 mm of rut depth. A target value of at least 1000 passes/mm was adopted to ensure adequate resistance to permanent deformation. Typical test specimens are illustrated in Figure 5.

In addition to the quantitative performance tests, aggregate coating and adhesion were qualitatively evaluated through static water immersion tests on loose mixtures. Samples were submerged in water for 16 h and subsequently examined to assess retained binder coverage on aggregate surfaces. Acceptable adhesion performance was defined as retention of at least 95% aggregate coating. Observations from these tests were used to supplement the mechanical and moisture susceptibility results.

All performance tests were conducted in accordance with applicable ASTM standards, and reported values represent the average of at least three replicate specimens for each test condition.

## 3. Results

This section presents the mixture design optimization outcomes and assesses whether the high-RAP half-warm repair mixture satisfies the predefined volumetric and mechanical performance targets. It evaluates the effects of asphalt binder content and additive dosage on density, VTM, Marshall stability, and flow in order to determine the OAC and establish a balanced mix composition suitable for low-temperature production and field repair conditions.

### 3.1. Mixture Design Optimization

**Figure 6:**
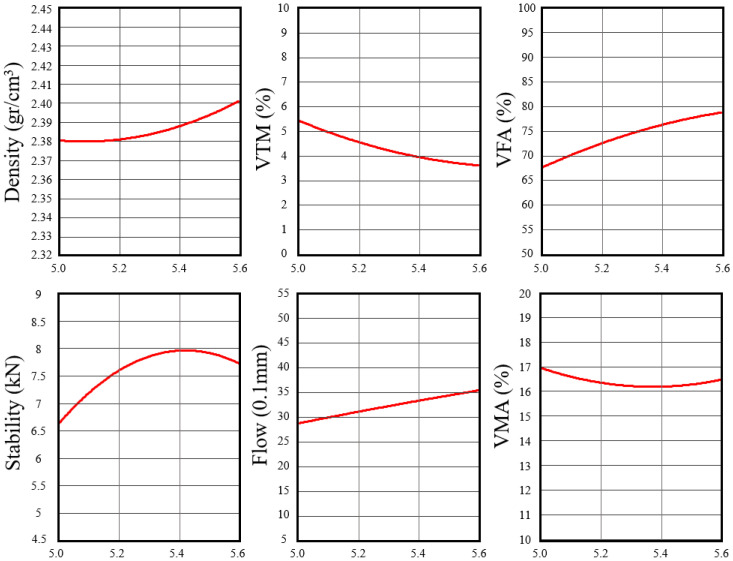
Marshall test properties versus asphalt content (%).

The Marshall mix design results were used to identify the OAC for the half-warm RAP mixture. Marshall test results for asphalt binder contents of 5.0%, 5.3%, and 5.6%. The corresponding trends in bulk density, VTM, Marshall stability, and flow are illustrated in Figure 6.

As the asphalt binder content increased from 5.0% to 5.6%, the mixture exhibited a progressive increase in bulk density accompanied by a reduction in VTM, indicating improved aggregate coating and compaction. Marshall stability increased with binder content and reached a maximum value of 7.86 kN at 5.3%, before decreasing slightly at 5.6%. This reduction at higher binder content suggests a weakening of the internal aggregate structure due to excess binder. Flow values increased over the same range, reflecting increased mixture ductility with higher binder content.

At a binder content of 5.3%, the mixture achieved a VTM of 4.1%, a VFA of 74.6%, and a VMA of 16.3%, all of which fall within the specified limits for asphalt repair materials. This binder content also corresponded to the highest Marshall stability among the evaluated mixtures. Based on these combined volumetric and mechanical results, a total asphalt binder content of 5.3% by mixture weight was identified as the OAC and was adopted for subsequent performance evaluation.

**Figure 7:**
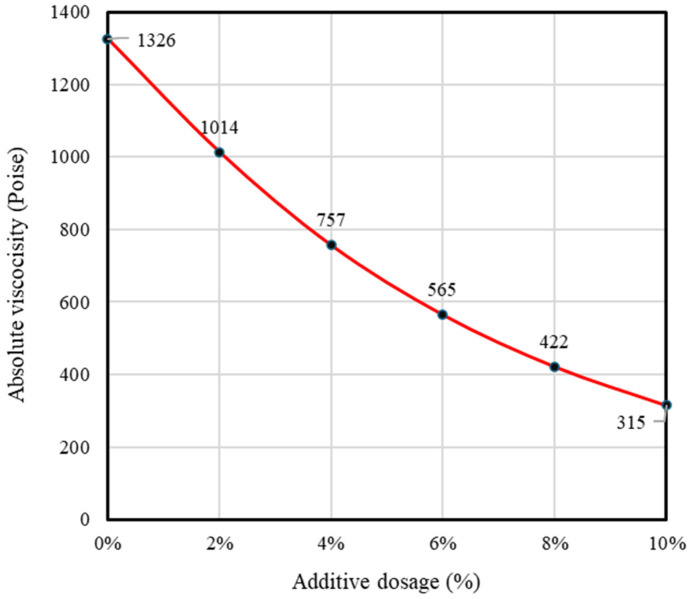
Viscosity–dosage relationship for rejuvenator determination based on the absolute viscosity of aged RAP binder at 60 °C.

To characterize the rheological condition of the aged RAP binder and establish a stable binder system, absolute viscosity testing of the recovered binder was conducted at 60 °C. The recovered RAP binder exhibited a significantly higher viscosity (3920.3 Pa·s) than the virgin binder (10.064 Pa·s), indicating severe oxidative ageing and limited workability at reduced production temperatures. Based on the target total binder content (5.3 wt.%) and the binder mass balance analysis, the required virgin binder content was determined as 2.1 wt.% of the mixture. The rejuvenator demand was then estimated using the viscosity–dosage relationship obtained from absolute viscosity testing (Figure 7), in which additive content was correlated with the reduction in binder viscosity. By interpolating the measured RAP binder viscosity within this empirical curve to reach a workable viscosity range for half-warm mixing, the required rejuvenator content was determined to be approximately 17.9% of the binder phase. This value was subsequently converted to a total mixture basis using the calculated virgin binder content, yielding a final dosage of 0.378 wt.% (rounded to 0.38 wt.%). The dosage was therefore selected based on rheology-guided workability considerations derived from the measured viscosity–dosage relationship of the binder system.

With the rejuvenator dosage held constant, the effect of the low-temperature additive on mixture volumetric and mechanical performance was evaluated using trial mixtures containing 0.07%, 0.10%, and 0.15% additive by total mixture mass, with the corresponding Marshall results summarized in Table 7. The RAP contents reported in Table 5 relate to the preliminary Marshall trial stage used for OAC determination, whereas the final mix formulation (Table 8) reflects the reconstructed composition after fixing the rejuvenator and optimal additive dosage and normalizing all components to 100 wt.%, yielding a finalized RAP content of 73.8 wt.% in the optimized mixture.

**Figure 8:**
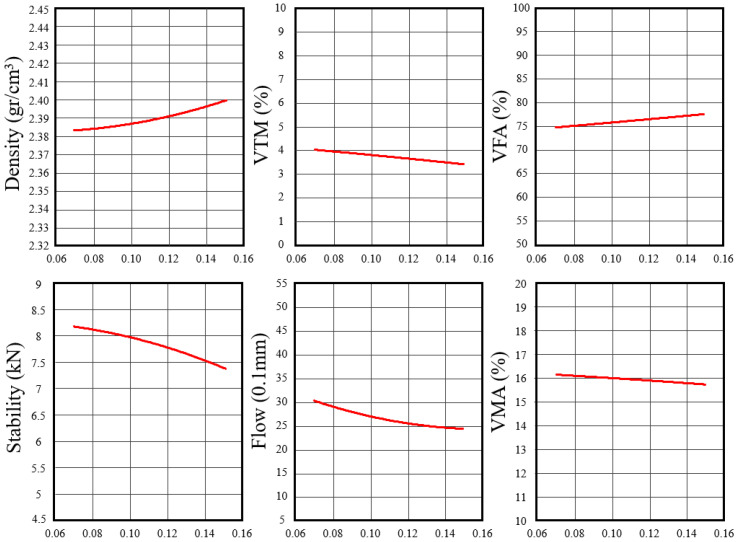
Marshall test properties versus low-temperature additive content.

As shown in Figure 8, increasing the low-temperature additive content resulted in a slight increase in bulk density and a corresponding reduction in VTM, which decreased from 4.1% at 0.07% additive to 3.4% at 0.15%. However, Marshall stability decreased from 8.14 kN at 0.07% additive to 7.48 kN at 0.15%, indicating diminishing mechanical benefit at higher additive dosages. Flow values also decreased from 3.07 mm to 2.43 mm, suggesting an increase in mixture stiffness, likely associated with the solid constituents of the additive, including recycled polymer and gilsonite. Although VFA increased with additive content, all mixtures remained within acceptable design limits.

Among the evaluated dosages, the mixture containing 0.07% low-temperature additive exhibited the most favorable balance of volumetric and mechanical properties, achieving the highest Marshall stability and a VTM closest to the design target of approximately 4%. Higher additive content did not result in additional performance benefits and would increase material usage. Accordingly, 0.07% by total mixture weight was selected as the optimal low-temperature additive content for the final mix formulation.

### 3.2. Performance Evaluation of Maintenance Material

**Figure 9:**
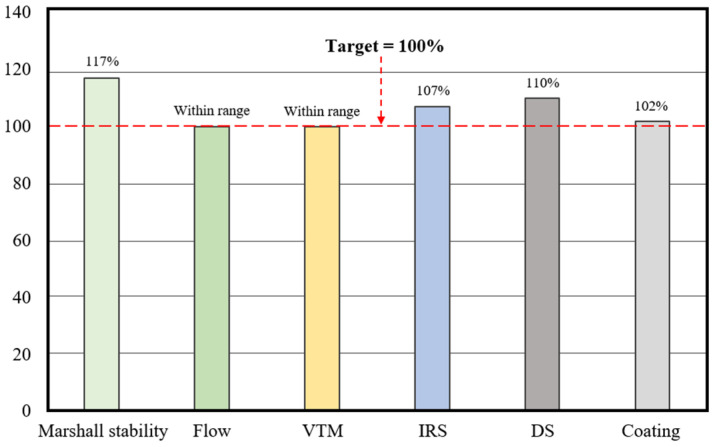
Performance vs. target for HWMA mix.

The performance of the optimized RAP–HWMA mixture incorporating 0.07% low-temperature additive was evaluated using a series of laboratory tests representative of the functional requirements of asphalt maintenance materials. Mechanical and volumetric properties were assessed using the Marshall method, while durability was evaluated through moisture susceptibility and rutting resistance tests. The Marshall test results and the corresponding performance trends are illustrated in Figure 9.

#### 3.2.1. Mechanical and Volumetric Performance

The optimized RAP–HWMA mixture demonstrated strong mechanical performance and temperature resilience. At 60 °C, it achieved an average Marshall stability of 5.84 kN (±0.15 kN), exceeding the project requirement of 5.0 kN and more than doubling the ASTM D1559 minimum of 2.5 kN for cold patch materials. The corresponding flow value was 3.6 mm, comfortably within the recommended range of 2.0–4.0 mm, indicating a favorable balance between stiffness and flexibility. These results suggest the mixture can bear early traffic loads without risking brittle failure or permanent deformation.

Similarly, the mixture exhibited a VTM of 7.0 ± 0.3% and a VFA of approximately 65%. This elevated air void level was intentionally maintained to allow for post-placement densification in the field and to mitigate the risk of binder bleeding. For comparison, a virgin control mixture yielded a lower VTM of 4.5 ± 0.2%, aligning with conventional dense-graded mix specifications but offering less flexibility for in-field densification. Overall, the RAP-based HWMA mixture achieved a well-balanced volumetric structure suited to patching conditions.

Workability was assessed qualitatively using a predefined rubric based on key indicators: ease of handling without excessive force, absence of visible clumping, no observable binder drain-down, and successful compaction to target density using standard Marshall effort (50 blows per face). The mixture satisfied all these criteria across trials, indicating that it could be handled and compacted effectively under low-temperature, small-batch production scenarios.

To further examine the temperature sensitivity of the mixture, compaction was performed at 50 °C, 70 °C, and 90 °C—temperatures chosen to bracket the practical HWMA production domain. This range reflects binder viscosity behavior (as per AASHTO T316 [39]) and operational limits relevant to field patching, where asphalt emulsions are often discouraged from being heated beyond ~85 °C. Within this framework, 60–80 °C is considered an emulsion-compatible range suitable for low-energy repairs, while 90–100 °C represents an upper subrange that facilitates coating and compaction without exceeding HWMA thresholds.

**Figure 10:**
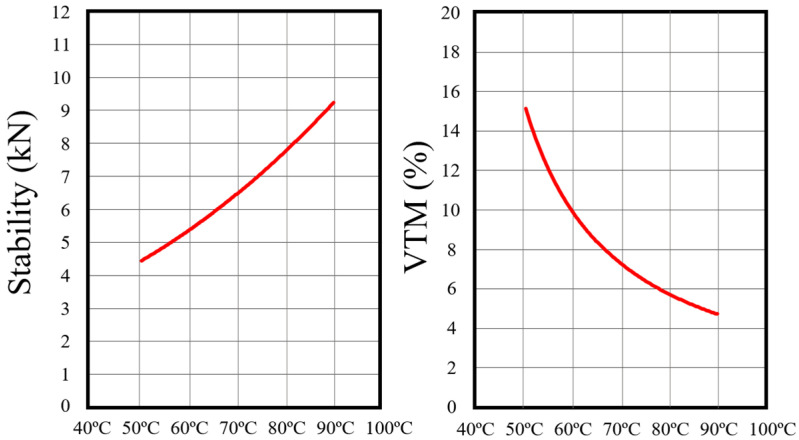
Effect of mixing temperature on RAP patch mix performance.

The results of this temperature sensitivity evaluation are summarized in Figure 10. At 50 °C, the mixture failed to meet critical performance benchmarks, with a VTM of 15.2% and a stability of just 4.50 kN—indicating inadequate binder activation and poor aggregate interlock. Performance improved markedly at 70 °C, where VTM dropped to 6.8% and stability increased to 6.50 kN, both within specification targets. At 90 °C, the mixture achieved its best outcomes, with VTM further reduced to 4.8% and Marshall stability rising to 9.20 kN. These results demonstrate that increased production temperature within the HWMA range significantly enhances compaction and mechanical integrity, though adequate performance can still be achieved at 70 °C, supporting flexibility in field application under varying thermal conditions.

#### 3.2.2. Durability and Deformation Resistance

The quantitative durability and deformation resistance results of the optimized mixture are summarized in Table 9. Moisture susceptibility was assessed by measuring Immersion Retained Stability (IRS) following 48 h of water immersion. The RAP mixture retained 80% of its original Marshall stability, surpassing the standard acceptance threshold of ≥75%. This outcome indicates adequate resistance to moisture-induced weakening. Additionally, the mixture’s resistance to stripping was assessed according to ASTM D2489 [40], achieving a binder coating of 99%, which exceeds the typical requirement of 95%. This high level of performance was confirmed, as conditioned specimens showed no evidence of stripping, and aggregate surfaces remained uniformly coated with no signs of binder separation. The results suggest that the mixture maintains structural cohesion under prolonged moisture exposure, a critical attribute for materials intended for application in wet or water-retentive environments, such as potholes.

DS testing was conducted at 60 °C to evaluate the mixture’s resistance to permanent deformation under repeated loading. The RAP mixture withstood 1100 passes/mm before reaching a rut depth of 1 mm, exceeding the project performance threshold of 1000 passes/mm. This result indicates a high level of rutting resistance, characteristic of heavy-duty asphalt materials. The test outcome demonstrates the mixture’s capacity to maintain structural integrity at elevated service temperatures, where deformation is more likely to occur. No premature displacement or lateral flow was observed during testing, and the deformation rate remained stable across the loading cycle. These results indicate strong high-temperature mechanical performance of the optimized RAP mix under sustained loading conditions.

## 4. Discussion

### 4.1. Mechanistic Interpretation of Material Performance

The improved performance of the HWMA mixture containing approximately 73.8% RAP is primarily attributed to the combined use of two additives with complementary functions. The rejuvenator restores the aged binder’s workability and blending capacity, while the low-temperature additive enables compaction at reduced production temperatures and reinforces the mix to retain structural performance under loading.

The rejuvenator, composed of 57.4% aromatic process oil, targets the oxidative aging of RAP binder. Aging depletes the maltene fraction and promotes asphaltene agglomeration, transitioning the binder from a viscoelastic sol state toward a brittle gel. The recovered RAP binder in this study exhibited an absolute viscosity of 3920.3 Pa·s at 60 °C, confirming extensive hardening. The rejuvenator is expected to improve binder mobility and blending capacity of the aged RAP binder by restoring the maltene–asphaltene balance and enhancing diffusion within the oxidized binder matrix, which in turn facilitates coating and compaction under short mixing cycles typical of patching operations [17,41].

The low-temperature additive, comprising recycled PET (26.3%), gilsonite (34.2%), naphtha-based oil (13.2%), and rosin (26.3%), provides both workability and structural reinforcement. The oil fraction aids coating and mixing at lower temperatures, while gilsonite enhances mastic stiffness and deformation resistance. The PET is expected to contribute additional rigidity through dispersed polymer reinforcement, and the rosin improves binder–aggregate adhesion, particularly under wet conditions. This dual-function strategy addresses a key challenge in high-RAP, low-temperature mixtures: softening agents alone can facilitate compaction but often weaken rutting resistance. By combining softening and stiffening components, the mix achieves workability without compromising mechanical integrity.

Experimental results support this interpretation. Among the trial dosages, the 0.07% low-temperature additive yielded the highest Marshall stability. Increasing the dosage to 0.10% and 0.15% did not improve compaction and slightly reduced stability, suggesting that once sufficient workability is achieved, further additives may begin to shift the mastic balance away from optimal strength development.

The optimized mixture (total binder 5.3%, including rejuvenator; low-temperature additive 0.07%) achieved a Marshall stability of 5.84 kN and dynamic stability of 1100 passes/mm at 60 °C. The high rutting resistance reflects the combined stiffness of the RAP-rich aggregate skeleton and the gilsonite-enhanced mastic. Moisture durability was also strong, with an IRS of 80% and binder coating coverage of 99%. The low standard deviations observed in Marshall stability (±0.15 kN) and volumetric parameters indicate stable mixture behavior despite the high RAP content, supporting the robustness of the optimized formulation. These outcomes are consistent with the expected role of rosin in enhancing binder–aggregate adhesion in high-RAP systems, although no direct interfacial or microscopic characterization was conducted in this study.

The effect of production temperature on performance reflected the thermomechanical response of the system. At lower temperatures, elevated binder viscosity restricted aggregate mobility and densification, resulting in higher air voids and lower structural integrity. At 50 °C, the mix exhibited 15.2% VTM and 4.5 kN Marshall stability. At 70 °C, voids decreased to 6.8% and stability increased to 6.5 kN, while 90 °C further improved compaction (VTM 4.8%) and strength (stability 9.2 kN). The additive sensitivity analysis (Table 9) indicates that increasing the low-temperature additive from 0.07 to 0.15 wt.% reduced Marshall stability from 8.14 kN to 7.48 kN while only marginally decreasing VTM (4.1% to 3.4%), demonstrating diminishing structural benefit beyond the optimal dosage and a progressive stiffening of the mastic phase. Expressed on a binder-mass basis, the optimal dosage of 0.07 wt.% of the mixture corresponds to approximately 1.3 wt.% of the total binder (5.3 wt.%). This level is consistent with dosages reported for concentrated modifier-type additives in recycled asphalt systems, where optimal performance is typically achieved at approximately 1.0–2.5 wt.% of binder, such as 1.5 wt.% for nano-modified RAP binders [42] and 2.5 wt.% for biopolymer-modified binders [40]. Unlike bulk rejuvenating oils, which rely on dilution and therefore require higher dosages, the composite additive used in this study functions through combined workability enhancement and structural reinforcement, explaining the low but effective optimal content.

To quantify this trend, first-order relations were fitted linking the three variables (temperature, *VTM*, and *stability*) within the tested domain, and a first-order relationship was developed in which mixing temperature influences early strength primarily through its effect on compacted *VTM*. Over the tested range (50–90 °C), *VTM* declined approximately linearly with temperature:

(2) $$\hat{VTM}=27.13-0.26\cdot T.$$

This suggests a reduction of about 0.26 percentage points in *VTM* per 1 °C increase. The fit was strong within the tested domain (R^2^ = 0.89). However, the 50 °C condition produced markedly higher voids than the other points and is therefore treated as a boundary case representing insufficient workability, not a basis for extrapolation.

Marshall stability also decreased with increasing *VTM*, as described by

(3) $$\hat{Stability}=9.08-0.306\cdot VTM.$$

This relationship supports the use of *VTM* as a primary structural indicator of early load-bearing capacity in the high-RAP HWMA system. Combining the two equations shows that *stability* gains with increasing temperature arise mainly from improved compaction (i.e., lower voids), though some additional benefit—particularly at higher temperatures—may result from improved coating, binder mobilization, and additive activation.

Based on these findings, 70 °C was the lowest temperature that met all performance criteria in the lab. To account for field variability, including heat loss during transport and inconsistent compaction, a conservative lower production temperature of 60 °C is recommended. Performance improvements beyond 70 °C were modest and do not justify the additional energy demand for most applications. Accordingly, a practical production temperature window of 60–100 °C is proposed, with the upper end (80–100 °C) preferred in colder weather or for larger repair areas. This range balances mechanical reliability with energy efficiency and supports field deployment of the mix under diverse operational conditions. These relationships were derived from three discrete temperature conditions (50, 70, and 90 °C) and are presented as descriptive trends within the experimentally tested domain, without extrapolation beyond the investigated temperature range. Furthermore, binder-level compatibility, phase separation behavior, and polymer dispersion were not directly evaluated; therefore, the proposed mechanisms are interpreted based on mixture-scale performance trends rather than microscopic verification.

### 4.2. Comparison with Prior Research and Existing Technologies

The performance of the developed mixtures represents a significant advancement over conventional repair materials. While standard CMA patches exhibit initial Marshall stabilities in the range of 2–4 kN [43], the mixture achieved an immediate stability of 5.84 kN, with a flow value of ~3.6 mm, indicating a good balance of stiffness and flexibility. Indicating the mixture possesses adequate flexibility to resist brittle fracture without being overly plastic. Furthermore, much of the existing research has focused on WMA with modest RAP fractions (typically 20–40%) or high-RAP mixtures produced at HMA temperatures [44]. For instance, Yousefi et al. [22] reported that incorporating 50% RAP in WMA mixtures significantly influences cracking and rutting performance depending on the type of WMA additive, highlighting the challenge of maintaining balanced performance at high RAP contents. The DS value of 1100 passes/mm is comparable to values reported for some polymer-modified HMA base courses and significantly exceeds the typical performance of rejuvenated RAP mixtures found in other studies [45,46]. This study demonstrated that it is technically feasible to incorporate approximately 73.8% RAP while producing the mixture at substantially lower temperatures (60 to 100 °C) than those conventionally used in asphalt production [43,47], showing that high RAP content and reduced mixing temperatures can be jointly achieved through a tailored additive strategy.

### 4.3. Practical Implications and Significance

This study demonstrates that producing high-RAP asphalt mixtures at 60–100 °C supports the development of half-warm patching materials that combine the rapid application of cold mixes with the structural performance of hot-mix asphalt. This approach offers practical benefits for pavement maintenance by reducing reapplication frequency, lowering lifecycle costs, and minimizing traffic disruptions. The mixture can be produced either by pre-mixing RAP with tailored additives at a plant or by on-site recycling using portable equipment, allowing flexibility across diverse repair scenarios. With approximately 75% RAP utilization and reduced production temperatures (60–100 °C), the proposed system decreases the demand for virgin aggregates and binder while lowering energy consumption and associated greenhouse gas emissions, as lower-temperature asphalt technologies and recycled material incorporation are widely reported to reduce environmental impacts compared to conventional hot-mix asphalt production [2,48].

However, several limitations warrant further study. First, while the mix performed well in controlled settings, field conditions such as low ambient temperature, wind, and surface moisture can compromise compaction and early strength. A practical lower-temperature threshold should be established as a function of environmental variables and pavement thickness, along with field protocols such as pre-heating or staged placement to ensure performance. Second, the study used a single RAP source; since RAP properties vary widely, future research should establish dosage adjustment guidelines based on binder viscosity or PG grade across multiple sources. Third, durability was confirmed using single-cycle immersion, but field conditions involve repeated wet–dry and freeze–thaw cycles. Multi-cycle and aged-state tests are needed to assess long-term durability and cracking resistance.

Finally, broader adoption will require economic and environmental evaluation. Life-cycle cost analysis (LCCA) and life-cycle assessment (LCA) should be conducted to quantify the full cost–carbon trade-offs of implementing this material at scale. Future work should therefore include (i) field validation under real-world conditions, (ii) RAP variability studies for dosage optimization, (iii) calibration of performance models for design use, and (iv) integrated LCCA–LCA to support agency decision-making.

## 5. Conclusions

This study evaluated the feasibility of a high-RAP half-warm asphalt mixture for pothole repair using a dual-additive system, demonstrating the integration of a rejuvenator and a low-temperature additive to enable low-temperature production while maintaining practical engineering performance. Laboratory results showed that the mixture could be produced and compacted at 60–100 °C and that the measured performance indicators (Marshall stability, flow, IRS, and dynamic stability) satisfied the predefined acceptance criteria established for asphalt repair applications:

- The optimized mix (73.8% RAP, total binder 5.3 wt.% including 0.38 wt.% rejuvenator and 0.07 wt.% low-temperature additive) was established through a sequential Marshall-based design and binder mass balance approach.
- The mixture achieved a Marshall stability of 5.84 kN (±0.15), flow of 3.6 mm, VTM of 7.0% (±0.3), IRS of 80%, and dynamic stability of 1100 passes/mm at 60 °C, satisfying the predefined performance criteria for asphalt repair applications.
- Additive optimization showed that increasing the low-temperature additive from 0.07 to 0.15 wt.% reduced stability from 8.14 kN to 7.48 kN with only minor VTM reduction (4.1% to 3.4%), indicating diminishing mechanical benefit beyond the optimal dosage.
- Temperature sensitivity results demonstrated that stability increased from 4.50 kN at 50 °C to 9.20 kN at 90 °C, while VTM decreased from 15.2% to 4.8%, confirming that compaction efficiency governs early mechanical performance in the high-RAP HWMA system.
- A production temperature of 70 °C was the lowest laboratory condition meeting all performance targets, while a practical field range of 60–100 °C is recommended to balance workability, compaction, and energy efficiency.

Overall, the developed high-RAP HWMA composite demonstrates that 73.8 wt.% RAP incorporation and half-warm production (60–100 °C) can be simultaneously achieved through a rheology-guided rejuvenator design combined with a dosage-controlled composite additive strategy for practical pavement repair applications. Nevertheless, several limitations should be acknowledged, including the use of a single RAP source, laboratory-based evaluation, absence of long-term fatigue and cracking assessment, and lack of advanced binder-level rheological characterization. Future research should therefore include field validation, multi-source RAP calibration, and comprehensive rheological and fracture testing to further substantiate long-term performance.

## 6. Patents

Our method is patent-registered in South Korea: “Agents for Asphalt mixture and thereof manufacturing method and Asphalt mixture using the Agents and thereof manufacturing method” Registration No. 10-1732718, Korean Intellectual Property Office (KIPO), 2 May 2023.

## Figures and Tables

**Table 1:** Aggregate gradations for asphalt mixtures.

Sieve Size (mm)	Passing (%)	
	RAP	Virgin Aggregate
25	100	100
20	100	100
12.5	100	98.2
10	88.8	80.4
5	72.6	11.8
2.5	48.8	-
0.6	29.4	-
0.3	14.7	-
0.15	8.7	-
0.08	5.3	-

**Table 2:** Typical formulation of a composite additive.

Component	Proportion (%)
Recycled PET	16.4
Blown asphalt	16.4
Aromatic process oil	57.4
Rosin	8.2
SBS	1.6

**Table 3:** Typical formulation of a low-temperature additive.

Component	Proportion (%)
Recycled PET	26.3
Gilsonite	34.2
Naphtha process oil	13.2
Rosin	26.3

**Table 4:** Estimation of initial total asphalt content.

Classification	Results
$P_{b}$	5.3
a	60
b	34
c	6
X	0.20
F	0.50

**Table 5:** Trial mix compositions for OAC determination.

Component	5.0 wt.% Asphalt	5.3 wt.% Asphalt	5.6 wt.% Asphalt
Reclaimed material (RAP, aggregate portion)	71.3	71.1	70.8
Virgin aggregate (13 mm)	21.8	21.7	21.7
Filler	1.90	1.90	1.90
Aged asphalt content, AP (in RAP)	3.20	3.20	3.20
Virgin asphalt content (added binder)	1.80	2.10	2.40
Total	100	100	100

**Table 6:** Trial mix compositions for optimal additive content.

Component	Mix A (%)	Mix B (%)	Mix C (%)
RAP	73.8	73.8	73.8
Virgin aggregate (12.5 mm)	21.75	21.72	21.67
Rejuvenator	0.38	0.38	0.38
Binder (AP-5)	2.1	2.1	2.1
Filler	1.9	1.9	1.9
Low-temperature additive	0.07	0.1	0.15
Total	100	100	100

**Table 7:** Marshall test results by additive (mean ± SD, n = 3).

Additive Content (%)	Density (g/cm^3^)	VTM (%)	VFA (%)	Stability (kN)	Flow (0.1 mm)	VMA (%)
0.07	2.38 ± 0.001	4.1 ± 0.1	74.7 ± 0.3	8.14 ± 0.08	30.7 ± 1.53	16.2 ± 0.06
0.10	2.39 ± 0.003	3.7 ± 0.1	76.5 ± 0.5	8.08 ± 0.04	28 ± 1.73	15.9 ± 0.10
0.15	2.40 ± 0.005	3.43 ± 0.2	78.3 ± 1.4	7.48 ± 0.25	24.3 ± 2.08	15.6 ± 0.21

**Table 8:** Final mix formulation.

Component	Mix (%)
RAP	73.8
Virgin aggregate (12.5 mm)	21.75
Rejuvenator	0.38
Binder (AP-5)	2.1
Filler	1.9
Low-temperature additive	0.07
Total	100

**Table 9:** Durability and deformation resistance of the optimized mixture.

Parameter	Test Condition	Result
Retained Marshall Stability (IRS)	48 h water immersion	80%
Coating degree (ASTM D2489)	Static immersion	99%
Dynamic stability (DS)	Wheel tracking at 60 °C	1100 passes/mm

## Data Availability

Data will be made available on request.
